# Laparoscopic Management of Jejunal Perforation Caused by Toothpick Ingestion: A Case Report

**DOI:** 10.7759/cureus.103102

**Published:** 2026-02-06

**Authors:** Luis Francisco Llerena Freire, Cintya Anabel Llerena Ojeda, Wilson Alexander Atiaja Arias, Edwin Guillermo Bahamonde, Nicole Belen Castro Farez, Santiago Vinicio Mera Morales

**Affiliations:** 1 Digestive Surgery, Hospital das Clínicas, Faculdade de Medicina, Universidade de São Paulo, São Paulo, BRA; 2 General Surgery, Hospital de Especialidades Eugenio Espejo, Ministerio de Salud Pública, Quito, ECU; 3 Teaching, Facultad de Ciencias Médicas, Universidad Central del Ecuador, Quito, ECU; 4 Teaching, Pontificia Universidad Católica del Ecuador, Quito, ECU; 5 Medical Physics, Ministerio de Salud Pública, Guayaquil, ECU; 6 Teaching, Facultad de Ciencias Médicas, Universidad San Francisco de Quito, Quito, ECU

**Keywords:** acute abdomen, computed tomography, foreign body ingestion, jejunal perforation, laparoscopy, toothpick ingestion

## Abstract

Foreign body ingestion is a frequent clinical problem, although most ingested objects pass spontaneously through the gastrointestinal tract. Sharp and elongated foreign bodies, such as toothpicks, are associated with a higher risk of perforation and may present diagnostic challenges due to their radiolucent nature.

We report the case of a 62-year-old man who presented with a four-day history of abdominal pain, fever, nausea, and signs of peritoneal irritation. Laboratory tests revealed leukocytosis and elevated inflammatory markers. Contrast-enhanced abdominal computed tomography demonstrated a linear foreign body approximately 4 cm in length, oriented perpendicular to the jejunal axis, with focal bowel wall thickening and adjacent mesenteric inflammation, consistent with small-bowel perforation.

Urgent diagnostic laparoscopy was performed. Intraoperatively, minimal inflammatory fluid and localized fibrinopurulent plaques were observed. Two small jejunal perforations were identified, one of which contained a protruding wooden toothpick. The foreign body was removed laparoscopically, and primary enterorrhaphy was performed. The postoperative course was uneventful, with early return of bowel function and no infectious or surgical complications. The patient was discharged on postoperative day 4 and remained asymptomatic at follow-up.

This case highlights the importance of maintaining a high index of suspicion for gastrointestinal perforation following the ingestion of sharp foreign bodies. Computed tomography plays a central role in diagnosis, and in selected stable patients, laparoscopy provides a safe and effective approach for definitive management, allowing diagnosis, foreign body extraction, and bowel repair in a single minimally invasive procedure.

## Introduction

Foreign body ingestion is a common gastrointestinal emergency, particularly in pediatric populations, but it also occurs in adults, most often as an accidental event. In the majority of cases, ingested objects pass spontaneously through the gastrointestinal tract without the need for intervention. However, sharp or elongated foreign bodies are associated with a significantly higher risk of complications, including mucosal injury, obstruction, and perforation [1-3].

Toothpicks represent a particularly hazardous foreign body because they are sharp, rigid, and frequently radiolucent, which may delay diagnosis when standard imaging is inconclusive. Although gastrointestinal perforation related to foreign body ingestion occurs in less than 1% of cases, toothpicks are disproportionately represented among reported perforations due to their ability to penetrate the bowel wall [4-6]. Perforation most commonly occurs at sites of physiological narrowing, such as the ileocecal valve, while jejunal involvement is relatively uncommon and may present with nonspecific symptoms, mimicking other causes of acute abdomen [7,8].

When perforation is suspected, computed tomography plays a central role in diagnosis, allowing the identification of radiolucent foreign bodies and associated inflammatory changes and facilitating timely surgical decision-making [8]. Endoscopic retrieval is the preferred treatment for accessible foreign bodies; however, distal small-bowel involvement or established complications often necessitate operative management [9]. In hemodynamically stable patients, laparoscopy offers the advantages of minimally invasive surgery while allowing definitive diagnosis and treatment. We report a rare case of jejunal perforation caused by toothpick ingestion successfully managed by laparoscopic removal and primary enterorrhaphy.

## Case presentation

A 62-year-old man with a medical history of chronic ischemic heart disease (acute coronary syndrome in 2021, treated with coronary revascularization and maintained on aspirin and lipid-lowering therapy), gout treated with allopurinol, and subclinical hypothyroidism on levothyroxine presented to the emergency department with a four-day history of progressively worsening abdominal pain. The pain was initially epigastric and later became diffuse, associated with nausea, vomiting, and subjective fever. The patient did not recall ingesting any foreign body.

On admission, physical examination revealed abdominal tenderness with signs of peritoneal irritation. Vital signs were as follows: blood pressure 104/65 mmHg, heart rate 91 beats per minute, respiratory rate 17 breaths per minute, oxygen saturation 95% on room air, and temperature 36.5°C. Laboratory investigations demonstrated marked leukocytosis (white blood cell count 19,200/µL) with neutrophilia (86%) and elevated C-reactive protein (11 mg/L) (Table 1). 

**Table 1:** Laboratory investigations on admission

Parameter	Result	Reference range
White blood cell count (WBC)	19,200/µL	4,000-11,000/µL
Neutrophils	86%	40-75%
C-reactive protein (CRP)	11 mg/L	<5 mg/L
Hemoglobin	15 g/dL	13.5-17.5 g/dL
Platelet count	350,000/µL	150,000-450,000/µL
Serum creatinine	0.9 mg/dL	0.7-1.3 mg/dL
Blood urea nitrogen (BUN)	Not reported	7-20 mg/dL
Serum sodium	Not reported	135-145 mmol/L
Serum potassium	Not reported	3.5-5.1 mmol/L
Serum lactate	Not reported	0.5-2.0 mmol/L

**Figure 1:**
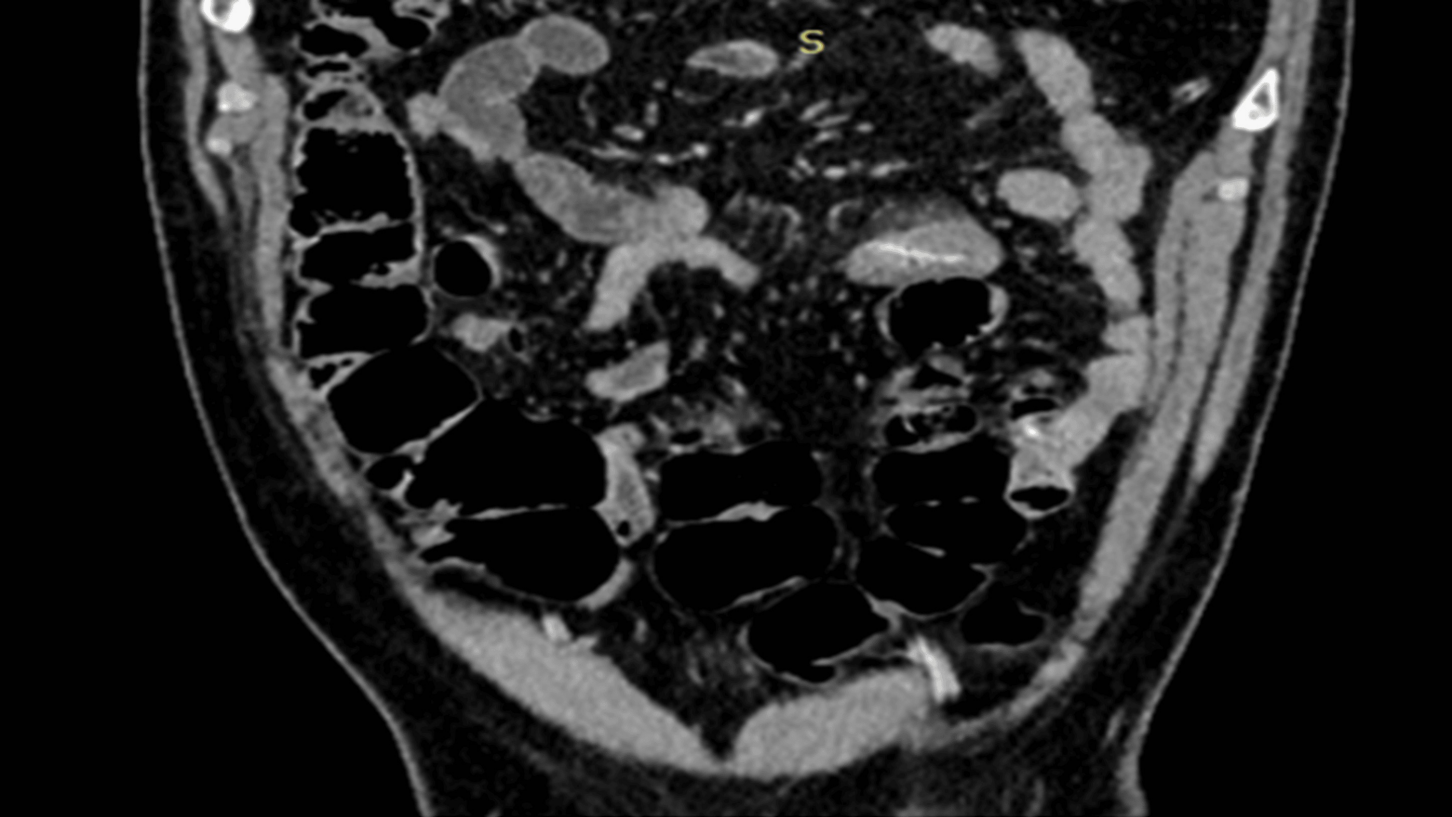
Contrast-enhanced computed tomography scan of the abdomen and pelvis showing a linear foreign body in the proximal jejunum (arrow) with surrounding bowel wall thickening and mesenteric fat stranding

**Figure 2:**
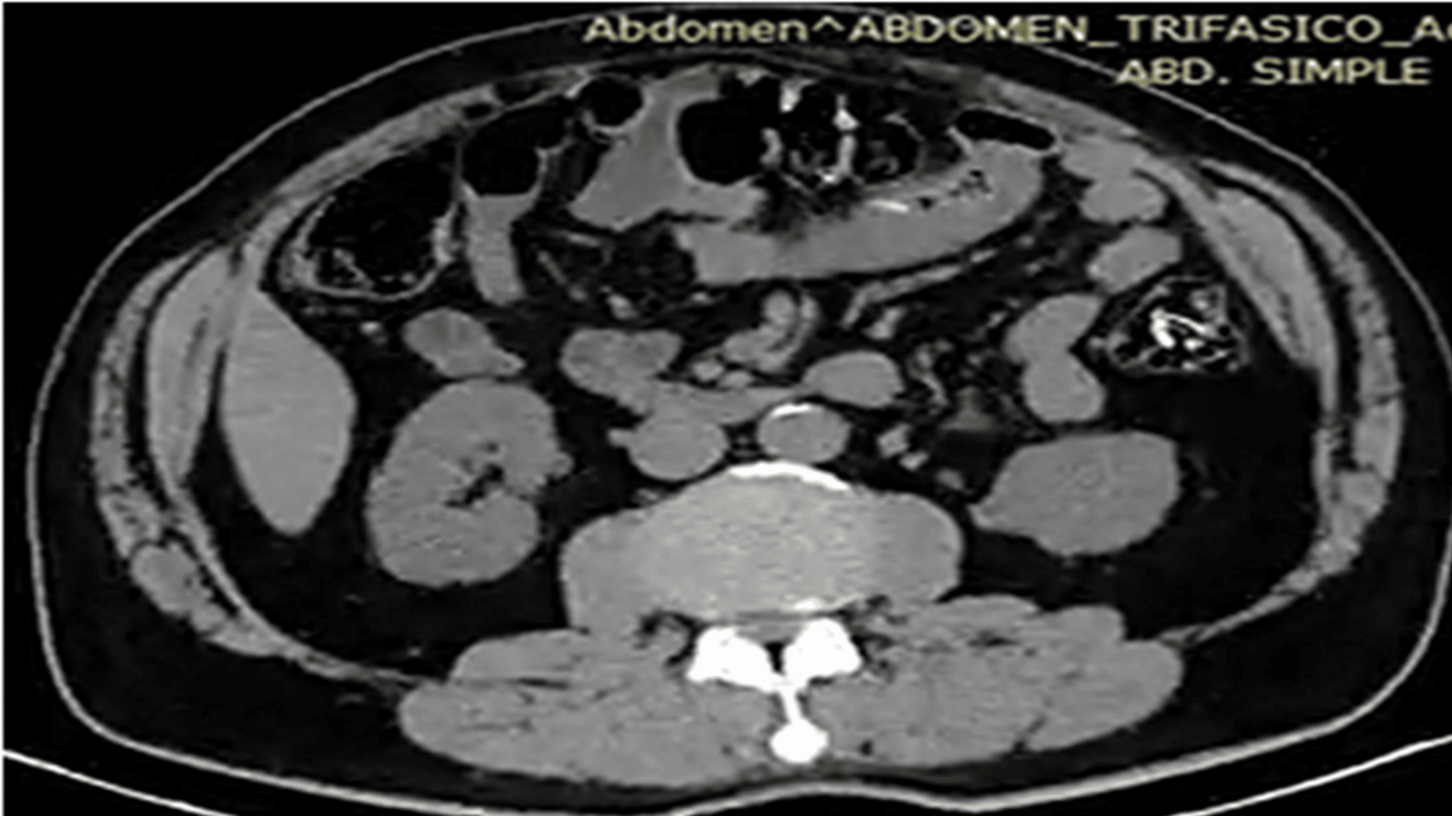
Coronal computed tomography view demonstrating the foreign body oriented perpendicular to the small-bowel axis with localized inflammatory changes

Contrast-enhanced abdominopelvic computed tomography revealed a focal inflammatory segment of the mid-proximal small bowel with circumferential wall thickening measuring up to 7.8 mm and surrounding mesenteric fat stranding. A linear foreign body approximately 4 cm in length was identified, oriented perpendicular to the jejunal axis, with imaging features consistent with small-bowel perforation (Figures 1-2). Based on the clinical presentation and radiological findings, a diagnosis of acute abdomen secondary to jejunal perforation by a foreign body was established, and urgent operative management was indicated.

**Figure 3:**
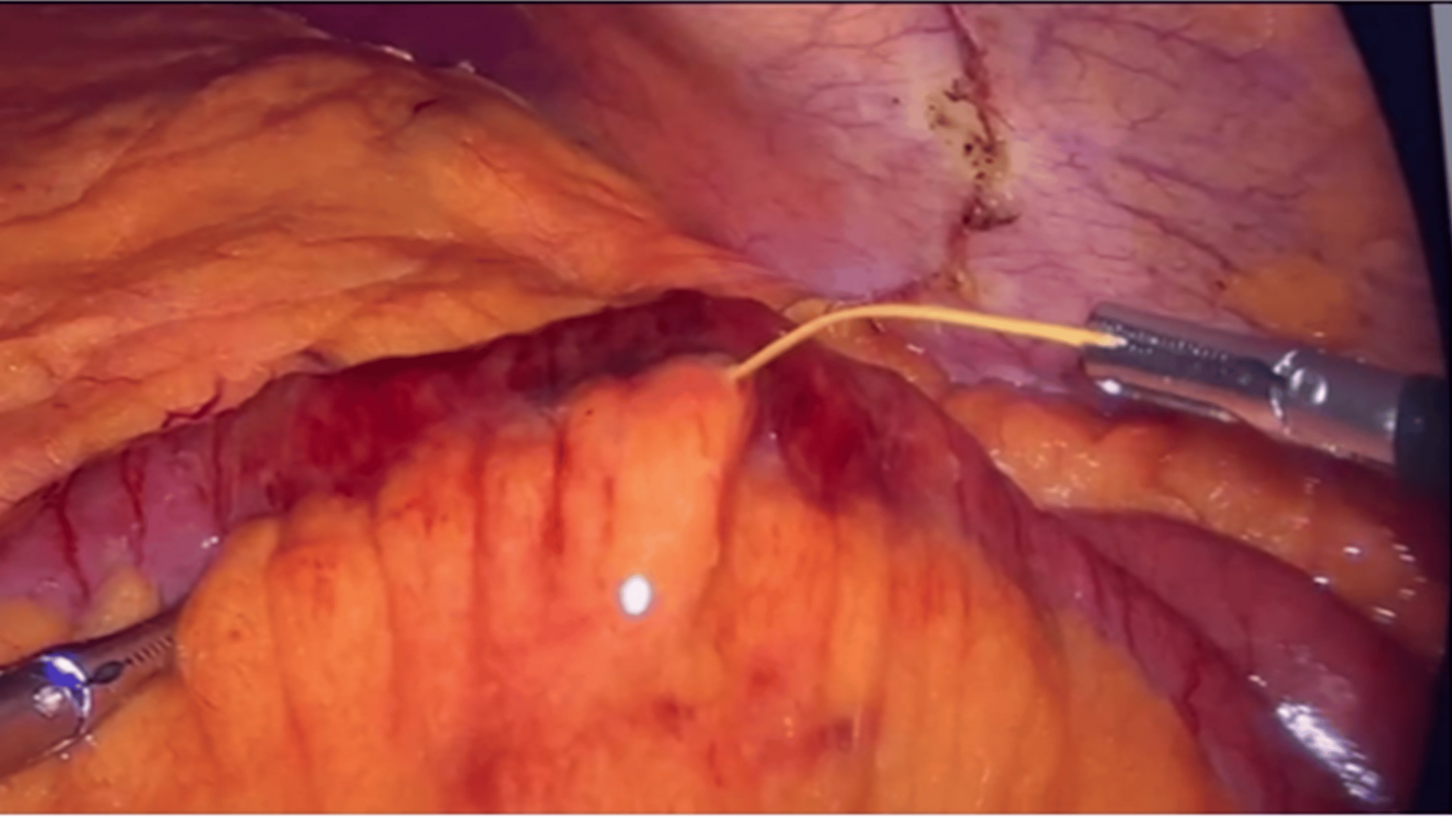
Laparoscopic view showing the extraction of the toothpick from the mesenteric-side jejunal perforation

The patient underwent diagnostic and therapeutic laparoscopy on the day of admission. Intraoperatively, a small amount of inflammatory peritoneal fluid and localized fibrinopurulent plaques over the jejunum were observed. Careful exploration identified two small perforations measuring approximately 2 mm in diameter: one on the antimesenteric border located 1.80 m distal to the ligament of Treitz and a second on the mesenteric border at 1.85 m from the ligament of Treitz. A wooden toothpick measuring approximately 5×0.2 cm was found protruding through the mesenteric-side perforation. After the removal of the foreign body, the surrounding bowel discoloration improved, suggesting preserved intestinal perfusion (Figure 3).

**Figure 4:**
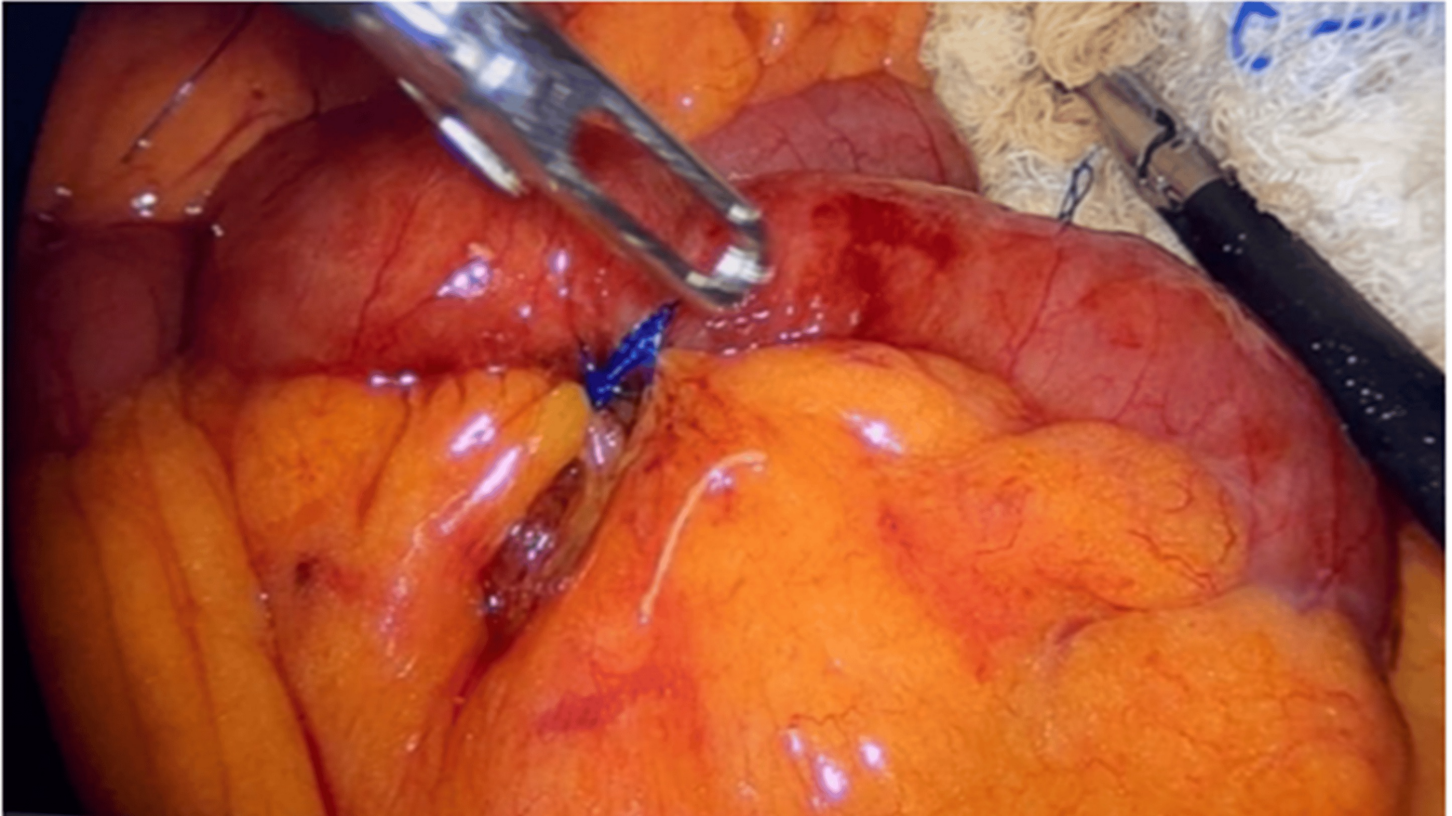
Laparoscopic primary repair (enterorrhaphy) of the jejunal perforation after foreign body removal

Primary laparoscopic enterorrhaphy was performed to close both defects. No bowel resection was required. A closed-suction drain was placed adjacent to the repair site. Postoperatively, the patient was managed with bowel rest until return of intestinal transit, followed by gradual advancement from clear liquids to a soft diet. His postoperative course was uneventful, with adequate pain control, minimal drain output, and progressive normalization of inflammatory markers (Figure 4).

The abdominal drain was removed, and the patient was discharged in stable condition on postoperative day 4, without intra-abdominal or wound-related complications (Clavien-Dindo grade I). At outpatient follow-up seven days after discharge, he remained asymptomatic with satisfactory recovery.

## Discussion

Foreign body ingestion is a frequent clinical problem, with the majority of ingested objects passing spontaneously through the gastrointestinal tract. Nevertheless, sharp and elongated foreign bodies, including fish bones, needles, and toothpicks, are associated with a significantly higher risk of impaction and complications [2,3,5]. Among these, toothpicks are particularly dangerous due to their rigidity, sharp ends, and frequent radiolucency, which contribute to delayed diagnosis and an increased likelihood of bowel wall penetration [5,7].

Gastrointestinal perforation secondary to foreign body ingestion is reported in fewer than 1% of cases; however, toothpicks are overrepresented among perforating objects [5,7]. Perforation typically occurs at anatomical narrowing points such as the ileocecal valve, while jejunal involvement is relatively uncommon. When the jejunum is affected, clinical presentation is often nonspecific, mimicking other causes of acute abdomen, and patients frequently do not recall ingestion, further complicating diagnosis [7,8].

Computed tomography is the imaging modality of choice when perforation is suspected, particularly in cases involving radiolucent foreign bodies. Computed tomography not only allows visualization of linear or punctiform foreign bodies but also identifies indirect signs of perforation, including bowel wall thickening, localized pneumoperitoneum, and mesenteric inflammatory changes [8]. In the present case, computed tomography findings of a linear foreign body oriented perpendicular to the bowel axis with surrounding inflammation were instrumental in establishing the diagnosis and expediting surgical management.

Endoscopic retrieval is considered the first-line treatment for accessible gastrointestinal foreign bodies and achieves high success rates when the object is located in the esophagus or stomach [6,9]. However, endoscopic management becomes limited once the foreign body has migrated beyond the reach of conventional endoscopy or when complications such as perforation have occurred. Surgical intervention is therefore indicated in the presence of peritoneal irritation, radiological evidence of perforation, or clinical deterioration despite conservative management [7,10].

Traditionally, laparotomy has been the standard surgical approach for small-bowel perforation. However, in selected hemodynamically stable patients without diffuse peritonitis, laparoscopy offers a safe and effective alternative. Minimally invasive surgery allows definitive diagnosis and treatment in a single procedure, with reduced surgical trauma, shorter hospital stay, and faster postoperative recovery compared with open surgery [3,6]. In the present case, laparoscopic exploration enabled the prompt identification of two small jejunal perforations, removal of the toothpick, and primary enterorrhaphy without the need for bowel resection, resulting in an uncomplicated postoperative course.

This case underscores several important clinical lessons. First, clinicians should maintain a high index of suspicion for gastrointestinal perforation when evaluating patients with an acute abdomen, even in the absence of a clear history of foreign body ingestion. Second, computed tomography is essential for timely diagnosis and operative planning in suspected cases involving sharp or radiolucent objects. Finally, laparoscopy should be considered a valuable therapeutic option in stable patients, providing definitive management with favorable outcomes when appropriate surgical expertise is available.

## Conclusions

Toothpick ingestion represents a high-risk form of foreign body ingestion because sharp and often radiolucent objects can cause gastrointestinal perforation and present with nonspecific clinical features, leading to diagnostic delay. Computed tomography plays a pivotal role in identifying radiolucent foreign bodies and associated signs of perforation, thereby guiding timely therapeutic decision-making.

When jejunal perforation is confirmed and the patient is hemodynamically stable without diffuse peritonitis, laparoscopic exploration allows definitive diagnosis and treatment in a single minimally invasive procedure. Laparoscopic foreign body extraction with primary enterorrhaphy is a safe and effective option that can result in rapid recovery and favorable outcomes when performed by experienced surgical teams.
